# Sensitivity of Food-Based Recommendations Developed Using Linear Programming to Model Input Data in Young Kenyan Children

**DOI:** 10.3390/nu13103485

**Published:** 2021-09-30

**Authors:** Karin J. Borgonjen-van den Berg, Jeanne H. M. de Vries, Prosper Chopera, Edith J. M. Feskens, Inge D. Brouwer

**Affiliations:** 1 Division of Human Nutrition and Health, Wageningen University, P.O. Box 17, 6700 AA Wageningen, The Netherlands; jeanne.devries@wur.nl (J.H.M.d.V.); pchopera@gmail.com (P.C.); edith.feskens@wur.nl (E.J.M.F.); inge.brouwer@wur.nl (I.D.B.); 2 Department of Nutrition Dietetics and Food Science, Faculty of Science, University of Zimbabwe, Mt Pleasant, Harare P.O. Box MP 167, Zimbabwe

**Keywords:** sensitivity analysis, linear programming, food-based dietary guidelines, 24 h dietary recall, consumption frequency, low- and middle-income countries

## Abstract

Food-based recommendations (FBR) developed using linear programming generally use dietary intake and energy and nutrient requirement data. It is still unknown to what extent the availability and selection of these data affect the developed FBR and identified problem nutrients. We used 24 h dietary recalls of 62 Kenyan children (4–6 years of age) to analyse the sensitivity of the FBR and problem nutrients to (1) dietary intake data, (2) selection criteria applied to these data and (3) energy and nutrient requirement data, using linear programming (Optifood©), by comparing a reference scenario with eight alternative scenarios. Replacing reported by estimated consumption frequencies increased the recommended frequencies in the FBR for most food groups while folate was no longer identified as a problem nutrient. Using the 10–90th instead of the 5–95th percentile of distribution to define minimum and maximum frequencies/week decreased the recommended frequencies in the FBR and doubled the number of problem nutrients. Other alternative scenarios negligibly affected the FBR and identified problem nutrients. Our study shows the importance of consumption frequencies for developing FBR and identifying problem nutrients by linear programming. We recommend that reported consumption frequencies and the 5–95th percentiles of distribution of reported frequencies be used to define the minimum and maximum frequencies.

## 1. Introduction

Healthy diets are vital to preventing undernutrition, micronutrient deficiencies and overnutrition which are still widespread public health problems [1]. While some progress has been made on decreasing the prevalence of undernutrition (including stunting and wasting), micronutrient deficiencies persist and the prevalence of overweight, obesity and diet-related non-communicable diseases due to malnutrition are increasing across the globe—rising the fastest in low-income countries [2,3]. Targeting poor diets is one of the major strategies to reverse malnutrition in all its forms and prevent related non-communicable diseases. However, the challenge is to move toward healthy diets that are notably more diverse with a greater proportion of micronutrient-dense foods [4]. 

Required changes towards healthy diets can be facilitated by food-based dietary guidelines (FBDG). FBDG are science-based recommendations intended for consumer information. These are used to inform the general population on how to compose a healthy diet that provides adequate amounts of foods and nutrients to prevent deficiencies and diet-related diseases. FBDG contain short evidence-based messages expressed in terms of foods to be consumed [5,6], often combined with visuals. The importance of developing FBDG per country for different age groups has been emphasised by the World Health Organisation (WHO) and the Food and Agriculture Organisation (FAO) since 1992 [7]. Still, currently only 93 out of 226 countries have officially endorsed FBDG by the government, including just 7 countries in Africa [8].

In the absence of national government-endorsed FBDG, food-based recommendations (FBR) have been developed to promote certain foods for specific purposes, regions, sex and age groups amongst others. In previous years, the linear programming approach was used to develop FBR in various African countries [9,10,11,12,13,14]. This approach uses information on existing food habits with the advantage that the developed FBR, if adopted, will improve nutrient intake with minimal deviation from the habitual diet while considering nutritional constraints such as energy requirements and price. Therefore, it is generally assumed that such developed recommendations will be acceptable and affordable for the targeted populations. In addition, problem nutrients—nutrients for which nutrient adequacy cannot be achieved using local foods available—can be objectively identified and can guide towards alternative additional strategies needed to fulfil nutrient adequacy [15].

Linear programming requires model input data such as energy and nutrient goals as well as dietary intake data. The model input data depend on the availability and selection of such data. Energy and nutrient goals depend on the source of requirement data, such as WHO/FAO, European Food Safety Authority (EFSA) or Institute of Medicine (IoM) [16,17,18,19]. In addition, other model input data, such as a list of commonly consumed foods, consumed amounts per food per day and minimum and maximum frequency of consumption per food are extracted from available local dietary intake data of the target population, often collected using the 24 h dietary recall method [20,21]. The number of participants, number of 24 h recalls per participant as well as availability of additional information per food (such as frequency of consumption) will affect the model input data. In addition to the availability of dietary intake data, model input data depends on selection criteria applied to the dietary intake data. Selection criteria are used to choose commonly consumed foods from all foods consumed as well as to define the minimum and maximum frequency per food, which determines the boundaries of the modelling. Different selection criteria are used in various studies and may affect the outcomes of the modelling and the resulting FBR and identified problem nutrients. 

Sensitivity of the developed FBR and of identified problem nutrients to these choices is often described only in general terms in the discussion of papers using linear programming [9,10,11,12,22,23,24,25,26], but rarely quantified by sensitivity analysis [9,23,25,26]. It is therefore unknown what effect the choice of dietary intake data, selection criteria and energy and nutrient requirement data have on the final results of linear programming. To determine the robustness of the developed FBR and the type and number of identified problem nutrients using linear programming, sensitivity analyses are needed. 

Using dietary intake data of Kenyan children 4–6 years of age, in this methodological paper we present the sensitivity of the developed FBR and the type and number of problem nutrients to (1) quality of dietary intake data, (2) selection criteria applied to dietary intake data and (3) energy and nutrient requirement data using linear programming. To address this sensitivity may be useful for the design of future linear programming studies in low- and middle-income countries.

## 2. Materials and Methods

### 2.1. Study Design

**Figure 1:**
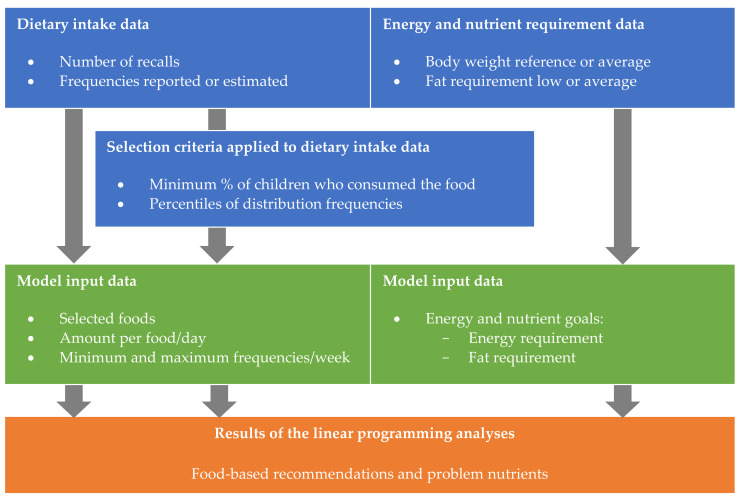
Illustration of the study design: to analyse the sensitivity of the developed FBR and type and number of problem nutrients to (1) quality of dietary intake data, (2) selection criteria applied to this data and (3) energy and nutrient requirement data using linear programming and 24 h dietary recalls of Kenyan children 4–6 years of age.

Optifood© was used to develop FBR and to identify problem nutrients using linear programming [27]. One reference scenario was compared with eight alternative scenarios. In each alternative scenario one aspect of the dietary intake data, selection criteria, or energy and nutrient requirement data was changed while maintaining other aspects unchanged (Figure 1). 

We used dietary intake data collected in a subsample of 112 randomly selected non-breastfed children 2–6 years of age, who were part of a larger randomised controlled double blind trial investigating the effectiveness of zinc-fortified drinking water on increasing zinc status [28]. The study was conducted in Kisumu West District, Nyanza province in Western Kenya, near Lake Victoria. Dietary intake data were collected over a period of 2 weeks in August 2014 during the pre-harvest season to evaluate dietary intake on population level. For the current linear programming study only the dietary intake data of children 4–6 years of age (*n* = 62) was used since these children constituted the largest age group in the dataset with similar nutrient requirements.

### 2.2. Dietary Assessment and Anthropometry

Two quantitative multiple pass 24 h recalls per child were conducted on non-consecutive days [21,29]. For the total population, recalls were evenly distributed over all days of the week and randomly assigned to well-trained interviewers who fluently spoke the local language. Details on the dietary assessment method as well as anthropometry are described elsewhere [23]. 

In summary, all foods and drinks consumed by the child the day before the interview (over a 24 h period) were listed. To assess amounts per food, ingredient and beverage, similar foods of comparable size were weighed or when the actual food was not available in the household, amounts were estimated in monetary value, volume, household measures or general sizes (small, medium, large). Frequency per food was reported as the number of days each food was consumed by the child over the previous 7 days. Conversion and waste factors were used to convert alternative amounts into grams. Standard recipes were composed when details of recalled dishes were missing. 

A food composition table was specifically compiled for this study, based on the national food composition table of Kenya [30], and supplemented with data from other food composition tables [31,32,33,34]. United States Department of Agriculture (USDA) retention factors [35] were applied to raw ingredients and foods to account for nutrient losses during preparation. The nutrient calculation program Compl-eat (version 1.0, Wageningen University, Wageningen, The Netherlands) including the compiled food composition table was used to calculate energy and nutrient intake per child per day. Average dietary intake, coefficient of variation and percentage children with an average intake below estimated average requirement (EAR derived from FAO/WHO recommended nutrient intakes (RNI) using conversion factors from IoM) [36], were calculated for energy and the nutrients of interest (using SPSS Statistics 25): total fat, total protein, calcium, iron, zinc, thiamin, riboflavin, niacin, vitamin B6, folate, vitamin B12, vitamin C and vitamin A [37]. 

Body weight and height were measured, and z-scores were calculated for height for age (HAZ) and BMI for age (BAZ) using WHO Anthro plus (version 3.2.2, www.who.int/childgrowth/software/en/ (accessed on 23 June 2017)). Stunting and thinness were defined as HAZ or BAZ less than −2 SD respectively. 

The effectiveness trial was registered at www.clinicaltrials.gov (NCT0216223) and approved by the Ethical Review Committee of Kenyatta National Hospital/Nairobi University (KNH-ERC/A/335) and ETH Zurich Ethical review committee (EK 2013-N-31). Before the start of the study, written informed consent was obtained from the head of the household and the caregiver on behalf of the child.

### 2.3. Determining Diet Models for Various Scenarios

For the reference scenario, model input data were defined using dietary intake data from both 24 h recalls per child. These model input data consisted of (1) a list of non-condiment foods reported by ≥3% of the children in one of the two recalls. Using 3% instead of the generally used 5% [9] allowed us to increase the number of foods in the modelling, as the variety of foods in the diet of our population was low; (2) median daily amount for each selected food for those consuming the food; (3) the minimum and maximum frequency of consumption per week for each food and (sub-) food group. Minimum and maximum frequency per week were defined as 5th and 95th percentiles, respectively, of the reported frequencies per food per week [25]. Reported frequencies of both recalls were included independently in the distribution estimation. Foods that were not present in one recall were assumed not to be consumed during the 7 days prior to that recall. All modelled diets had to meet the energy requirement which was calculated using the mean body weight of the target group and the FAO/WHO/United Nations University (UNU) algorithm for estimating energy requirements [16]. Nutrient goals were set as recommended nutrient intakes (RNI) defined by FAO/WHO [17,38,39] for the nutrients of interest. Since the fat requirement was defined as a range of 25–35% of energy (en%), in the reference scenario the average requirement of 30 en% was used. Low bioavailability for iron and zinc (5% and 15%, respectively) was assumed for an unrefined cereal-based diet with high levels of phytate. Energy and nutrient composition per 100 g of the selected foods were adopted from the compiled food composition table.

Eight alternative scenarios were defined to test the sensitivity of the developed FBR and the type and number of problem nutrients (Table 1). Per alternative scenario, one of the selection criteria used in the reference scenario was replaced by an alternative criteria. The first three alternative scenarios A, B and C were compared with the reference scenario to evaluate the impact of dietary intake data on FBR and problem nutrients. In the first alternative scenario A, the reported frequencies per food per week used in the reference scenario were replaced by estimated frequencies per food per child. These were based on the number of days the food subgroup appeared in the two recalls, converted to a frequency per week and the proportion of children that consumed the food. This latter method to estimate frequencies per food per week is commonly used to define model input data for Optifood^©^ [9,11,13,23,25]. In the second alternative scenario B only the first of the two 24 h recalls was used to define the model input data. The third alternative scenario C was a combination of scenario A and B where estimated frequencies per week were combined with only the first recalls. 

Alternative scenarios D, E and F were compared with the reference scenario to evaluate the impact of selection criteria applied to dietary intake data on FBR and problem nutrients. In the alternative scenario D, only non-condiment foods consumed by ≥10% of the children were selected in an attempt to stay closer to the average food pattern. In alternative scenario E, all non-condiment foods consumed were used to define the model input data, irrespective of how many children consumed these foods. In alternative scenario F minimum and maximum frequency per week for selected foods and food (sub)groups were narrowed and defined as 10th and 90th percentiles, respectively, of the reported frequencies per food per week to remain closer to the average food pattern. 

The last two alternative scenarios G and H were compared with the reference scenario to evaluate the impact of energy and nutrient requirement data on FBR and problem nutrients. In alternative scenario G energy requirements were estimated using the FAO/WHO/UNU algorithm including reference body weight instead of mean body weight as in the reference scenario [40]. In the alternative scenario H, the nutrient goal for fat was defined as the lower tail of the fat requirement (25 en%). 

### 2.4. Linear Programming Analyses

Linear programming analyses were performed in Optifood^©^, a linear programming approach to model realistic diets for target populations and to objectively identify problem nutrients [27]. For the reference scenario as well as the 8 alternative scenarios, 3 modules were run per scenario. 

Module I was run to ensure that the model input data were generating realistic and feasible diets. Module II was run to develop the best-optimised diet (draft FBR) reaching nutrient adequacy for as many nutrients as possible, limited by the minimum and maximum frequencies per week and the energy requirement. Module III was run to identify problem nutrients, nutrients that were unable to reach 100% RNI in the maximised diet. One maximised diet for each nutrient of interest was modelled and included the most nutrient dense foods within each food group to verify the highest possible nutrient intake of that nutrient. The draft FBR developed in module II as well as the problem nutrients defined in module III were compared between the alternative scenarios and the reference scenario. 

## 3. Results

### 3.1. Characteristics and Dietary Intake of the Study Population

Slightly more girls (*n* = 36) than boys (*n* = 26) were included in the dietary assessment study (Table 2). Body weight and height were measured in 60 out of 62 children, of whom 13 (22%) were stunted. The prevalence of stunting was higher in boys (*n* = 8) than in girls (*n* = 5) and no children were underweight. 

Dietary assessment included two 24 h dietary recalls per child with, on average, 8 days between the first and second recall and a total of 124 recalls. In both recalls, 86 different non-condiment food items were reported in the dietary recalls, of which 64 food items were reported by at least 3% of the children in at least one of the two recalls. The most commonly consumed foods were maize, tomato, onion, milk, vegetable oil and sugar (consumed by >80% of the children). Median consumption frequency was highest for vegetables with two types of vegetables consumed per day (Supplementary Table S1). The median energy intake was 1489 kcal/day (25–75th percentiles: 1172–1852 kcal/day). For seven nutrients the median intake was below the EAR for >50% of the children. Vitamin A, zinc and folate had the highest percentage of children below EAR (98%, 82% and 76% respectively). The within-person coefficient of variation for this population was highest for vitamins B12, A and C (104%, 98% and 90% respectively). Vitamin A, calcium and vitamin B12 had the highest between-person coefficient of variation (69%, 51% and 49% respectively) (Table 2). 

### 3.2. The Effects of Scenarios on FBR and Problem Nutrients

Developed draft FBR for the reference scenario consisted of added fats 7 times/week, dairy products 8 times/week, fruits 7 times/week, grains and grain products 21 times/week, legumes, nuts and seeds 4 times/week, meat, fish and eggs 7 times/week and vegetables 28 times/week (Table 3). Draft FBR in alternative scenarios were mainly affected when the reported frequencies were replaced by the estimated frequencies (scenario A including 2 recalls and scenario C including 1 recall). The recommended frequencies per week in the draft FBR increased for most food groups in both scenarios compared to the reference draft FBR. Furthermore, in scenario F, when the tails of the distribution of consumption frequencies per week of the foods and food (sub)groups were narrowed to the 10th and 90th percentiles, the recommended frequencies per week decreased for the fruits, meat, fish and eggs and vegetables food groups compared to the reference draft FBR, and the legumes, nuts and seeds food group was no longer included. The effects of the other alternative scenarios on the draft FBR were negligible. 

Problem nutrients in the reference scenario were folate (94% RNI), vitamin A (56% RNI) and zinc (86% RNI) (Table 4). The number of problem nutrients decreased from 3 to 2 when the reported frequencies were replaced by estimated frequencies in scenarios A and C, since folate was no longer identified as a problem nutrient (130% and 128% RNI respectively). Total fat did not reach the goal of 30 en% when only foods consumed by at least 10% of the children were included (scenario D) and when the 10th and 90th percentiles were used to define the minimum and maximum frequencies of consumption per week (scenario F). However, the fat content of the maximised diet remained within the requirement range of 25–35 en% (respectively 29 en% and 28 en%). Moreover, in the latter scenario (scenario F), the highest number of problem nutrients were identified including riboflavin, niacin, folate, vitamin B12, vitamin A, iron and zinc.

### 3.3. The Effects of Scenarios on Model Input Data

Only 37 out of 64 commonly consumed foods were included in the food list in the reference scenario. This is because the frequency of consumption per week was 0 in the 95th percentile for the 27 excluded foods (Table 5). Alternative scenario A, using estimated frequencies, contained the highest number of foods in the food list (*n* = 59), while scenario F, using the 90th percentile to define maximum frequencies per week, contained the lowest number of foods in the food list (*n* = 26). In scenario E, which used all 86 foods consumed, only 37 foods were included in the food list as the frequency of consumption of the excluded foods consumed by less than 3% of the children was 0 in the 95th percentile. This resulted in the model input data being identical to the reference scenario. The number of foods reported by at least 3% of the children decreased from 64 using two recalls to 50 using only the first recalls (scenarios B and C). From the 50 foods reported in the first recall, daily amount per food remained the same for 12 foods, increased for 18 foods and decreased for 20 foods compared to the daily amount per food using two 24 h recalls (Supplementary Table S2).

The minimum and maximum consumption frequencies per week used as model input data are shown in Supplementary Table S3. Nearly all recommended frequencies in the draft FBR were equal to the maximum consumption frequencies per week either defined as the 95th percentile (reference scenario) or the 90th percentile of distribution (scenario F). In addition, either minimum and/or maximum frequency per week increased for all food groups when estimated frequencies were used (scenarios A and C) compared to reported frequencies in the reference scenario. 

The estimated energy requirement in scenario G increased from 1256 kcal/day to 1427 kcal/day when reference body weight (19.2 kg) instead of the mean actual body weight (16.9 kg) was used to define the energy requirement of the target group. Due to the increased body weight and energy requirement, the absolute estimated protein requirement rose from 12 g to 13 g and the absolute fat requirement from 42 g to 48 g. When the fat requirement was defined as 25 en% (scenario H) instead of 30 en% in the reference scenario, the absolute fat requirement decreased from 42 g to 35 g. 

## 4. Discussion

To our knowledge, this is the first study that has investigated the sensitivity of FBR and of identified problem nutrients to the selection of dietary intake data, criteria and energy and fat requirements by linear programming. The sensitivity of the results of linear programming to the model input data is often mentioned, but rarely quantified [9,23,25,26]. 

Our study showed that the results of linear programming, i.e., draft FBR and type and number of problem nutrients, were most sensitive to the consumption frequencies and the percentiles defining minimum and maximum frequencies per week (Table 6). The draft FBR were most affected by the use of estimated frequencies (based on the presence in the 24 h dietary recalls) instead of reported frequencies. Estimated frequencies increased recommended frequencies of most food (sub)groups in the draft FBR. The number of problem nutrients increased from 3 to 7 when the 10–90th instead of 5–95th percentiles were used. The results of linear programming in our population were less sensitive to the number of recall days per child, criteria to define the food list and selected level of energy and fat requirement. 

To compare the alternative scenarios, we used the draft FBR including the most nutrient-dense foods available within the set of constraints generated in the linear programming analyses in this study (model II results), and not the final FBR (module III analysis). To develop realistic final FBR, recommendations per food (sub)group and food need to be tested and combined (module III analysis), requiring thorough knowledge about local food patterns and therefore the involvement of local experts and policymakers. However, this would introduce additional subjective decisions on the development of FBR, influencing the ability to attribute possible differences in the resulting FBR of the studied scenarios solely to changes in the model input data. 

The 24 h dietary recall method, used to assess intake, is the preferred method in low- and middle-income countries [21,42]. However, there are random and systematic errors related to this method, such as the memory of the participant, interviewer bias, portion size estimation and nutrient values in the food composition table. Although measurement errors were minimised as much as possible in this study, these errors may have affected the absolute values of dietary intake data, draft FBR and problem nutrients. However, as these errors were present in all scenarios, the comparison between the scenarios was assumed not to be affected.

Using estimated frequencies instead of reported frequencies increased the recommended frequencies of most food (sub)groups in the draft FBR. Estimated frequencies resulted in higher minimum and maximum frequencies per food and food (sub)group used as model input data. Minimum and maximum frequencies were defined using the distribution of consumption frequencies. The distribution of estimated frequencies was estimated per child, with possible frequencies of 7, 3.5 and 0 if the food was consumed on both days, 1 of the 2 days or not consumed, respectively. Distribution of reported frequencies was based on the frequencies of foods present in all 124 recalls with all possible frequencies between 0, if the food was not consumed on that day, and 7, if the food was consumed every day in the previous 7 days. Using estimated frequencies probably overestimated the maximum frequencies per food and food (sub)group, because the consumption of a food on both days may not necessarily reflect consumption on every day in the previous week. This overestimation of maximum (and not of minimum) consumption frequencies resulted in higher recommended frequencies in the draft FBR. The recommended frequencies may therefore be too high to be acceptable and affordable for the target population. 

Although reported frequencies are expected to be more accurate than estimated frequencies, we only asked respondents to report frequencies of the foods they consumed in the recall. Consumption frequencies of foods that were not consumed on one of the recall days were therefore lacking, assuming that these foods were not consumed at all. Therefore, reported frequencies probably underestimated the minimum frequencies of foods and food (sub)groups. This could have affected the scenarios comparing the reported frequencies with the estimated frequencies. However, as the draft FBR in the scenarios with reported frequencies were not limited by the minimum consumption frequencies, comparison of the draft FBR and problem nutrients was assumed to be unaffected. 

Although the missing consumption frequencies were not expected to affect the results in this study, they may affect results in future research. To overcome this, a propensity questionnaire could be added to the 24 h dietary recall to collect consumption frequencies of irregularly consumed foods. The propensity questionnaire enables the researcher to include irregularly consumed, nutrient-dense foods in the model, which may decrease the number of problem nutrients [43,44]. 

In our study population, the 5–95th instead of the 10–90th percentiles of distribution may be preferred to define minimum and maximum frequencies per week of foods and food (sub)groups to reach nutrient adequacy for as many nutrients as possible. Using the 10th and 90th percentiles resulted in draft FBR closer to the average food pattern, with the advantage that the population is asked to make less changes to their food patterns allowing for easier adoption of the developed FBR. However, the number of problem nutrients doubled compared to the 5–95th scenario. In order to still reach nutrient adequacy for the problem nutrients in the 10–90th scenario, alternative interventions that may be difficult to adopt by the population have to be considered. Using the 5–95th percentiles resulted in fewer problem nutrients and draft FBR still remained within the current local food patterns and may therefore be preferred to define minimum and maximum frequencies. 

The number of recall days per child influenced the model input data, but did not affect the results of linear programming in our population. Minimum and maximum frequencies per week of foods and food (sub)groups used as model input data were more affected by the number of recall days when estimated consumption frequencies were used instead of reported frequencies. In addition, the food list was affected by the number of recall days per child. Using 1 instead of 2 recalls decreased the number of different foods consumed as expected, for scenarios using reported as well as estimated consumption frequencies. Conversely, the number of different foods included in the model increased using 1 instead of 2 recall days. As all foods with a maximum reported frequency (in the 95th percentile) of 0 consumptions per week were excluded from the food list, foods consumed on less than 6 out of 124 recall days (using both recall days) or 3 out of 62 recall days (using only 1 recall day) were not included in the model. In the recalls of the first day, 50 different foods were consumed of which 44 were consumed on at least 3 recall days. The recalls of the second day included slightly more foods (52 foods), however less foods were consumed on 3 or more recall days (36 foods) compared to the recalls of the first day. Using both recalls, 27 out of 64 foods were excluded, because these were consumed on less than 6 recall days. Reported consumption frequencies of the foods that were included in the food list of the first recall but excluded in the food list of both recalls were low. Consequently, reported minimum and maximum frequencies per week of foods and (sub) food (sub)groups used as model input data were only slightly different when 1 recall day was used instead of 2. Although the number of different foods was affected by the number of recall days when using reported consumption frequencies, the draft FBR and the number and type of problem nutrients were not affected by the number of recall days per child. 

Assessing the habitual dietary intake of the population remains a challenge, especially in low- and middle-income countries, and results depend among others on the within- and between-person variation in dietary intake [45]. This variation is affected by many factors such as research area, season, age group and prevalence of overweight and undernutrition [46,47,48]. The present study was conducted in a rural area of Kenya with a prevalence of stunting of 23% in children under 5 years and a high prevalence of nutrient intake below the EAR, which is comparable with our study [49]. Within- and between-person variation in intake were higher for most nutrients in our population compared to the variation in intake of 6–11-year-old children in the NHANES study conducted in 2007–2008 [50]. This indicates that nutrient-dense foods are not consumed regularly (or daily) and not consumed by the whole population. This high variation may be related to the differences in poverty in our study area, where for example, expensive foods are only affordable for the relatively rich, contributing to the between-person variation, or can only be afforded irregularly, contributing to the within-person variation [50]. The relatively small number of children (*n* = 62) as well as the two dietary recalls per child only could have increased the estimated variation [50]. More recalls per child in a larger population will decrease the variation and tighten the distribution of consumption frequencies. In addition, the tightened distribution may reduce the differences in minimum and maximum frequencies between the scenarios. Smaller differences in the model input data will also reduce the differences in draft FBR and problem nutrients between the scenarios. Additional research with larger sample sizes and different target groups is needed to confirm the effect of variation on the results of linear programming.

## 5. Conclusions

In conclusion, our study shows that draft FBR and the type and number of identified problem nutrients are most sensitive to model input data related to frequency of consumption of foods and food (sub)groups. We recommend using reported consumption frequencies and collecting the frequency data of commonly as well as irregularly consumed foods to avoid over- or underestimation in dietary intake. To limit the number of problem nutrients, we suggest defining the minimum and maximum frequencies used as model input data by using the 5th and 95th percentile of the distribution. However, additional research is needed to test the eligibility of developed FBR using these percentiles. As the model input data based on the distribution of the frequencies may be affected by variation in diets, among others affected by a small sample size, the results of our study should be confirmed in other populations.

## Figures and Tables

**Table 1:** Reference scenario and alternative scenarios A-H used to define model input data for linear programming.

Model Input Data	Selection Criteria	Frequencies	
		Reported	Estimated
Amount per food/day	2 dietary recalls	Reference scenario	Scenario A
	1 dietary recall	Scenario B	Scenario C
Selected foods	≥3% of children consumed the food	Reference scenario	
	≥10% of children consumed the food	Scenario D	
	All foods consumed	Scenario E	
Min and max frequencies/week per food and food (sub)group	5–95th percentile	Reference scenario	
	10–90th percentile	Scenario F	
Energy requirement	Based on average body weight	Reference scenario	
	Based on reference body weight	Scenario G	
Fat requirement	30 en% (mean) and average body weight	Reference scenario	
	25 en% (low) and average body weight	Scenario H	

**Table 2:** Characteristics of the Kenyan children in the study population (*n* = 62) including median intake per day and coefficients of variation for energy and the nutrients of interest ^1^.

		Median ^1^	25–75th Perc ^1^	CV%wtn ^2^	CV%btn ^3^	% below EAR ^4^
**Background**						
Sex, girls	*n* (%)	36 (58)				
Age	Y	5.3	4.6–6.0			
						
**Anthropometrics** ^5^						
Body weight	kg	16.9	15.5–18.4			
Height for age ^6^	z-score	−1.1	−1.9–0.4			
Stunted ^6^	N	13				
BMI for age ^6^	z-score	0.0	−0.6–0.6			
Underweight ^6^	N	0				
						
**Dietary intake of nutrients** ^7^						
Energy	kcal/d	1489	1172–1852	29.8	22.4	34
Protein	g/d	35.8	28.3–46.5	37.9	23.7	2
Fat	g/d	39.4	29.7–54.4	59.1	15.4	40
Thiamin	mg/d	0.78	0.58–1.11	51.4	22.5	18
Riboflavin	mg/d	0.49	0.36–0.70	54.6	36.7	52
Niacin	mg/d	5.05	4.03–6.38	50.1	0	68
Vitamin B6	mg/d	0.64	0.52–0.91	53.1	0	21
Folate	ug/d	112	74–159	62.5	32.3	76
Vitamin B12	ug/d	0.88	0.48–1.57	104.2	48.8	58
Vitamin C	mg/d	29.8	18.5–43.1	90.2	31.0	37
Vitamin A (RAE)	ug/d	95.5	49.1–150.0	98.3	69.2	98
Calcium	mg/d	511	300–669	68.6	50.8	48
Iron	mg/d	10.6	8.8–14.4	48.7	31.8	63
Zinc	mg/d	5.26	4.04–7.10	48.2	24.0	82

^1^ Values indicate median and 25–75th percentile unless indicated otherwise. ^2^ Within-person coefficient of variation. ^3^ Between-person coefficient of variation. ^4^ EAR: Estimated average requirement [36]. ^5^ Anthropometry was measured in 60 children. ^6^ Children were classified as stunted or underweight if their HAZ or BAZ respectively were less than −2 SD according to WHO child growth standards (<60 months) and WHO reference 2007 (>61 months) [40,41]. ^7^ Average of 2 recalls. N: number of children; Y: years.

**Table 3:** Draft FBR in frequency per week for the reference scenario and alternative scenarios defined in Optifood module 2 for Kenyan children, 4–6 years of age.

	Reference Scenario ^1^	Scenario A Est Freq ^2^	Scenario B Rp Freq ^3^ 1 Recall	Scenario C Est Freq ^2^ 1 Recall	Scenario D ≥ 10% Cons ^4^	Scenario E All Foods	Scenario F 10–90th Perc ^5^	Scenario G Ref Weight ^6^	Scenario H 25 en% Fat
Food group ^7^	Number of daily amounts per week								
Added fats	7	4	6	4	7	7	7	7	5
Added sugars	0	4	1	7	0	0	4	0	0
Bakery and breakfast cereals ^8^	0	0	0	0	--	0	--	2	2
Dairy products	8	11	12	14	7	8	7	8	8
Fruits	7	7	7	10	7	7	2	7	7
Grains and grain products	21	12	19	11	21	21	21	22	22
Legumes, nuts and seeds ^8^	4	7	4	3	3	4	--	4	4
Meat, fish and eggs	7	11	5	14	7	7	3	7	7
Starchy roots and other starchy plant foods ^8^	--	3	0	3	--	--	--	--	--
Vegetables	28	32	30	35	28	28	24	28	28

^1^ Reference scenario: 2 recalls, reported frequencies, selected foods consumed by ≥3% of the children, frequencies selected from 5th and 95th percentile of distribution, energy requirement based on average body weight and 30 en% fat requirement. ^2^ Est freq: Estimated frequencies. ^3^ Rp freq: Reported frequencies. ^4^ ≥10% cons: Foods selected that are consumed by at least 10% of the children. ^5^ 10–90th perc: Minimum frequencies/week selected from the 10th percentile of distribution and maximum frequencies/week selected from the 90th percentile of distribution. ^6^ Ref weight: Energy requirement based on reference body weight of the target group (4–6 years). ^7^ See Supplementary Table S2 for more details on classification of foods. ^8^ --: not included in the model.

**Table 4:** Identified problem nutrients as % of RNI in a maximised diet per nutrient for reference scenario and alternative scenarios for Kenyan children, 4–6 years of age.

	Maximised Diet								
	Reference Scenario ^1^	Scenario A Est Freq ^2^	Scenario B Rp Freq ^3^, 1 Recall	Scenario C Est Freq ^2^, 1 Recall	Scenario D ≥10% Cons ^4^	Scenario E All Foods	Scenario F 10–90th Perc ^5^	Scenario G Ref Weight ^6^	Scenario H 25 en% Fat
Nutrient	% RNI								
Protein	371	450	411	466	364	371	286	356	371
Fat (en%) ^7^	33	51	36	35	**29** ^9^	33	**28** ^9^	30	33
Thiamin	220	209	282	230	216	220	177	235	220
Riboflavin	134	188	179	215	115	134	**94** ^8^	136	134
Niacin	103	103	122	118	102	103	77 ^8^	106	103
Vitamin B6	177	183	196	207	177	177	149	192	177
Folate	**94** ^8^	130	**96** ^8^	128	**86** ^8^	**94** ^8^	**52** ^8^	**96** ^8^	**94** ^8^
Vitamin B12	110	205	173	285	101	110	**86** ^8^	111	110
Vitamin C	196	296	221	438	195	196	110	196	196
Vitamin A	**56** ^8^	**72** ^8^	**69** ^8^	**84** ^8^	**53** ^8^	**56** ^8^	**28** ^8^	**56** ^8^	**56** ^8^
Calcium	136	225	229	342	126	136	103	136	136
Iron	123	136	135	140	119	123	**97** ^8^	129	123
Zinc	**86** ^8^	**76** ^8^	**89** ^8^	**79** ^8^	**83** ^8^	**86** ^8^	**66** ^8^	**92** ^8^	**86** ^8^

^1^ Reference scenario: 2 recalls, reported frequencies, selected foods consumed by ≥3% of the children, frequencies selected from the 5th and 95th percentile of distribution, energy requirement based on average body weight and 30 en% fat requirement. ^2^ Est freq: Estimated frequencies. ^3^ Rp freq: Reported frequencies. ^4^ ≥10% cons: Foods selected that are consumed by at least 10% of the children. ^5^ 10–90th perc: Minimum frequencies/week selected from the 10th percentile of distribution and maximum frequencies/week selected from 90th percentile of distribution. ^6^ Ref weight: Energy requirement based on reference body weight of the target group. ^7^ Fat content of the maximised diets are shown in en%. ^8^ With FBR unable to reach 100% RNI. ^9^ With FBR unable to reach 30 en% fat, but within requirement of 25–35 en%.

**Table 5:** Number of foods consumed and included in the food list per scenario for Kenyan children, 4–6 years of age.

		Consumed	In Food List ^1^
Scenario		Number of Foods	
Reference scenario ^2^		64	37
Scenario A:	Estimated frequencies	64	59
Scenario B:	1 recall	50	44
Scenario C:	1 recall, Est freq ^3^	50	48
Scenario D:	≥10% consumed ^4^	33	33
Scenario E:	All foods consumed	86	37
Scenario F:	10–90th percentile ^5^	64	26

^1^ Food is included in food list when the frequency of consumption >0 in the 95th percentile (or 90th percentile in scenario F). ^2^ Reference scenario: 2 recalls, reported frequencies, selected foods consumed by ≥3% of the children, frequencies selected from 5th and 95th percentile of distribution. ^3^ Est freq: Estimated frequencies. ^4^ ≥10% cons: Foods selected that are consumed by at least 10% of the children. ^5^ 10–90th percentile: Minimum frequencies/week selected from the 10th percentile of distribution and maximum frequencies/week selected from the 90th percentile of distribution.

**Table 6:** Main changes in draft FBR and problem nutrients for Kenyan children 4–6 years of age caused by changes in dietary intake data, selection criteria and energy and nutrient requirement data.

	Reference Scenario ^1^	Scenario A Est Freq ^2^	Scenario B Rp Freq ^3^ 1 Recall	Scenario C Est Freq ^2^ 1 Recall	Scenario D (break) ≥ 10% Cons ^4^	Scenario E All Foods	Scenario F 10–90th Perc ^5^	Scenario G Ref Weight ^6^	Scenario H 25 en% Fat
**Draft FBR** ^7^	Frequencies per week	Changes in frequencies and problem nutrients compared to the reference scenario							
Added fats	7	**−**		**−**					**−**
Dairy products	8	**+**	**+**	**+**					
Fruits	7		Negligible	**+**	Negligible	None	**−**	Negligible	Negligible
Grains and grain products	21	**−**		**−**					
Legumes, nuts and seeds	4	**+**					**−**		
Meat, fish and eggs	7	**+**		**+**			**−**		
Vegetables	28	**+**		**+**			**−**		
Added sugars	0	**+**	**+**	**+**			**+**		
Starchy roots and other starchy plant foods ^8^	--	**+**		**+**					
Bakery and breakfast cereals	0							**+**	**+**
**Problem nutrients**									
Folate	**●**		**●**		**●**	**●**	**●**	**●**	**●**
Vitamin A	**●**	**●**	**●**	**●**	**●**	**●**	**●**	**●**	**●**
Zinc	**●**	**●**	**●**	**●**	**●**	**●**	**●**	**●**	**●**
Riboflavin							**●**		
Niacin							**●**		
Vitamin B12							**●**		
Iron							**●**		

**−** Frequency decreased compared to reference scenario. **+** Frequency increased compared to reference scenario; **●** Identified problem nutrient. ^1^ Reference scenario: 2 recalls, reported frequencies, selected foods consumed by ≥3% of the children, frequencies selected from the 5th and 95th percentile of distribution, energy requirement based on average body weight and 30 en% fat requirement. ^2^ Est freq: estimated frequencies. ^3^ Rp freq: reported frequencies. ^4^ ≥10% cons: foods selected that are consumed by at least 10% of the children. ^5^ 10–90th perc: minimum frequencies/week selected from the 10th percentile of distribution and maximum frequencies/week selected from the 90th percentile of distribution. ^6^ Ref weight: energy requirement based on the reference body weight of the target group. ^7^ See Supplementary Table S2 for more details on classification of foods. ^8^ --: not included in the model.

## Data Availability

The data supporting the reported results are available on request from the corresponding author.

## Supplementary Materials

The following are available online at https://www.mdpi.com/article/10.3390/nu13103485/s1. Table S1: Median consumption frequencies per food group per week. Table S2: Median daily amount per food consumed by > 3% of the Kenyan children 4–6 years of age, calculated from 2 recalls compared with 1 recall. Table S3: Minimum and maximum number of daily amounts per week required for linear programming in the reference scenario and 5 alternative ‘dietary intake data’ and ‘selection criteria’ scenarios for Kenyan children, 4–6 years of age.
