# Supplementary material for: Sensitivity of Food-Based Recommendations Developed Using Linear Programming to Model Input Data in Young Kenyan Children
Source: Nutrients. 2021 Sep 30;13(10):3485. doi: 10.3390/nu13103485 (PMC8541147; doi:10.3390/nu13103485)
Supplement: Supplementary file 1 [file nutrients-13-03485-s001.zip › Supplementary Table S1.pdf]

Supplementary Table S1. Median consumption frequencies per food group per week

Food group[1]	Consumption frequencies /week[2]
Added fats	7
Added sugars	7
Bakery & breakfast cereals	0
Dairy products	7
Fruits	0
Grains & grain products	10
Legumes, nuts & seeds	0
Meat, fish & eggs	1
Starchy roots & other starchy plant foods	0
Vegetables	14

[1] See Supplementary Table S2 for more details on classification of foods

[2] Based on 124 24-hour dietary recalls
