# Supplementary material for: Sensitivity of Food-Based Recommendations Developed Using Linear Programming to Model Input Data in Young Kenyan Children
Source: Nutrients. 2021 Sep 30;13(10):3485. doi: 10.3390/nu13103485 (PMC8541147; doi:10.3390/nu13103485)
Supplement: Supplementary file 1 [file nutrients-13-03485-s001.zip › Supplementary Table S2.pdf]

Supplementary Table S2. Median daily amount per food consumed by >3% of the Kenyan children 4-6 years of age, calculated from 2 recalls compared with 1 recall

Food name	2 recalls	1 recall	Food name	2 recalls	1 recall
	Amount per food (g/day)[1]			Amount per food (g/day)[1]	
**Added fats**			**Legumes, nuts & seeds**		
Fat	10	7	Beans black, dried[2]	48	--
Oil	24	19	Beans cocoa rose, dried	35	31
**Added sugars**			Beans mung, dried	61	71
Sugar brown	39	37	Beans red, dried	29	29
Sugar white	25	23	Beans white, dried	42	77
**Bakery & breakfast cereals**			Peas cow, dried	50	50
Bread white	74	64	Groundnut, fresh[3]	68	--
Bread white Supaloaf	89	89	Groundnut, boiled[3]	108	--
**Beverages**			**Meat, fish & eggs**		
Chocolate powder drinking[3]	22	--	Egg, baked	36	25
**Dairy products**			Catfish boiled	26	30
Milk cow whole, Brookside[2]	56	--	Fulu fish, fried	22	14
Milk cow whole, Brookside boiled	83	103	Mud fish, boiled[3]	59	--
Milk cow sour	237	222	Mud fish, fried[3]	26	--
Milk cow whole, fresh	222	308	Nile perch fish, boiled	15	15
Milk cow whole, boiled	94	98	Nile perch fish, fried[3]	36	--
**Fruits**			Tilapia fish, boiled[2]	62	--
Mango ripe	64	64	Tilapia fish, fried[2]	32	--
Avocado	93	129	Omena Dagaa, dried, boiled	14	13
Banana ripe	152	152	Omena Dagaa, dried, fried	18	20
Juice lemon	4	4	Omena Dagaa, boiled	35	35
Orange[3]	46	--	Omena Dagaa, fried	19	16
Papaya	37	34	**Starchy roots & other starchy plant foods**		
**Grains & grain products**			Plantain	83	83
Flour wheat Tropicana fortified	16	21	Flour cassava	23	14
Flour wheat white	15	19	Potatoes Irish[3]	58	--
Rice white	57	49	Potatoes sweet	172	172
Flour fingermillet	54	55	**Vegetables**		
Flour maize white whole	128	128	Onion bulb red, fried	4	4
Flour maize white[2]	108	--	Onion stem, boiled	2	3
Flour maize yellow	107	124	Onion stem, fried	4	3
Flour sorghum	27	23	Tomato, boiled	11	13
Maize grains white, dried	98	106	Tomato, fried	16	15
Maize grains yellow, dried	91	93	Sukuma wiki, fried	24	22
Maize, roasted	87	88	Cabbage, fried	98	122
			Cowpea leaves, boiled	38	22
			Okra, boiled	3	2
			Okra, fried[2]	7	--

[1] Values are median daily amounts of the raw or dried edible portions per food
[2] Food not reported in the first recalls
[3] Food consumed by <3% of the children in the first recalls and therefore not included in food list
