# Supplementary material for: Sensitivity of Food-Based Recommendations Developed Using Linear Programming to Model Input Data in Young Kenyan Children
Source: Nutrients. 2021 Sep 30;13(10):3485. doi: 10.3390/nu13103485 (PMC8541147; doi:10.3390/nu13103485)
Supplement: Supplementary file 1 [file nutrients-13-03485-s001.zip › Supplementary Table S3.pdf]

Supplementary Table S3 Minimum and maximum number of daily amounts per week required for linear programming in the reference scenario and 5 alternative 'dietary intake data' and 'selection criteria' scenarios for Kenyan children, 4-6 years of age

	Reference Scenario[1]		Scenario A Est freq[2]		Scenario B Rp freq[3], 1 recall		Scenario C Est freq[2], 1 recall		Scenario D ≥10% cons[4]		Scenario E All foods		Scenario F 10th-90th perc[5]	
	min	max	min	max	min	max	min	max	min	max	min	max	min	max
Food (sub)group	Number of daily amounts/week													
**Added fats**	**0**	**7**	**4**	**11**	**0**	**7**	**0**	**7**	**0**	**7**	**0**	**7**	**3**	**7**
Butter, ghee, margarine (unfortified)	0	7	0	7	0	7	0	7	0	7	0	7	0	7
Vegetable oil (unfortified)	0	7	0	7	0	7	0	7	0	7	0	7	0	7
**Added sugars**	**0**	**7**	**4**	**11**	**1**	**7**	**7**	**7**	**0**	**7**	**0**	**7**	**3**	**7**
**Bakery & breakfast cereals[6]**	**0**	**3**	**0**	**4**	**0**	**3**	**0**	**7**	**--**	**--**	**0**	**3**	**--**	**--**
**Dairy products**	**0**	**8**	**0**	**11**	**0**	**12**	**0**	**14**	**0**	**7**	**0**	**8**	**0**	**7**
Fluid or powdered milk (fortified)[6]	--	--	0	4	0	3	0	7	--	--	--	--	--	--
Fluid or powdered milk (non-fortified)	0	8	0	11	0	9	0	14	0	7	0	8	0	7
**Fruits**	**0**	**7**	**0**	**7**	**0**	**7**	**0**	**14**	**0**	**7**	**0**	**7**	**0**	**2**
Vitamin A source fruit	0	7	0	7	0	7	0	7	0	7	0	7	0	2
Vitamin C-rich fruit[6]	--	--	0	7	0	5	0	7	--	--	--	--	--	--
**Grains & grain products**	**4**	**22**	**11**	**28**	**4**	**24**	**7**	**35**	**4**	**22**	**4**	**22**	**5**	**21**
Enriched/fortified grains and products, whole or refined	0	7	0	7	0	7	0	7	0	7	0	7	0	3
Refined grains and products, unenriched/unfortified	0	7	0	11	0	7	0	14	0	7	0	7	0	4
Whole grains and products, unenriched/unfortified	2	21	7	21	1	23	7	28	2	21	2	21	4	16
**Legumes, nuts & seeds[6]**	**0**	**4**	**0**	**11**	**0**	**4**	**0**	**7**	**0**	**3**	**0**	**4**	--	--
Cooked beans, lentils and peas[6]	0	3	0	11	0	4	0	7	0	3	0	3	--	--
Nuts, seeds and unsweetened products[6]	0	1	0	4	--	--	--	--	--	--	0	1	--	--

Supplementary Table S3 Cont.

	Reference Scenario[1]		Scenario A Est freq[2]		Scenario B Rp freq[3], 1 recall		Scenario C Est freq[2], 1 recall		Scenario D ≥10% cons[4]		Scenario E All foods		Scenario F 10th-90th perc[5]	
	min	max	min	max	min	max	min	max	min	max	min	max	min	max
Food (sub)group	Number of daily amounts/week													
**Meat, fish & eggs**	**0**	**7**	**0**	**11**	**0**	**5**	**0**	**14**	**0**	**7**	**0**	**7**	**0**	**3**
Eggs[6]	0	1	0	4	0	1	--	--	0	1	0	1	--	--
Fish without bones[6]	0	4	0	7	0	4	0	7	0	4	0	4	--	--
Small, whole fish, with bones	0	3	0	7	0	4	0	7	0	3	0	3	0	3
**Starchy roots & other starchy plant foods[6]**	**--**	**--**	**0**	**4**	**0**	**1**	**0**	**7**	--	--	**--**	**--**	--	--
**Vegetables**	**2**	**28**	**11**	**32**	**2**	**30**	**7**	**35**	**2**	**28**	**2**	**28**	**5**	**24**
Other vegetables	0	21	4	21	0	28	0	28	0	21	0	21	3	21
Vitamin A source dark green leafy vegetables	0	4	0	7	0	5	0	7	0	4	0	4	0	3
Vitamin C-rich vegetables	0	6	0	11	0	6	0	14	0	6	0	6	0	4

[1] Reference scenario: 2 recalls, reported frequencies, selected foods consumed by ≥3% of the children, frequencies selected from the 5th and 95th percentile of distribution, energy requirement based on average bodyweight and 30 en% fat requirement

[2] Est freq: Estimated frequencies

[3] Rp freq: Reported frequencies

[4] ≥10% cons: Foods selected that are consumed by at least 10% of the children

[5] 10th-90th perc: Minimum frequencies/week selected from the 10th percentile of distribution and maximum frequencies/week selected from the 90th percentile of distribution

[6] --: not included in the model
